# Socio-demographic and sexual practices associated with HIV infection in Kenyan injection and non-injection drug users

**DOI:** 10.1186/s12889-018-5100-y

**Published:** 2018-01-30

**Authors:** Valentine Budambula, Charles Matoka, James Ouma, Aabid A. Ahmed, Michael F. Otieno, Tom Were

**Affiliations:** 1 Department of Environment and Health Sciences, Technical University of Mombasa, P. O. Box 90420-80100, Mombasa, Kenya; 2 Department of Applied Sciences, Technical University of Mombasa, P. O. Box 90420-80100, Mombasa, Kenya; 3 Department of Environmental Health, University of Kabianga, P. O. Box 2030-20200, Kericho, Kenya; 4 Bomu Hospital, P.O Box 95683-80106, Mombasa, Kenya; 5 Department of Medical Laboratory Sciences, Kenyatta University, P. O. Box 43844-00100, Nairobi, Kenya; 6 Department of Medical Laboratory Sciences, Masinde Muliro University of Science and Technology, P.O. Box 190-50100, Kakamega, Kenya

**Keywords:** HIV infection, Injection drug users (IDUs), Non-IDUs, Non-drug users, Socio-demographic and sexual risk practices

## Abstract

**Background:**

Substance use is increasingly becoming prevalent on the African continent, fueling the spread of HIV infection. Although socio-demographic factors influence substance consumption and risk of HIV infection, the association of these factors with HIV infection is poorly understood among substance users on the African continent. The objective of the study was to assess socio-demographic and sexual practices that are associated with HIV infection among injection drug users (IDUs), non-IDUs, and non-drug users (DUs) at an urban setting of coastal Kenya.

**Methods:**

A cross-sectional descriptive study was conducted among 451 adults comprising HIV-infected and -uninfected IDUs (*n* = 157 and 39); non-IDUs (*n* = 17 and 48); and non-DUs (*n* = 55 and 135); respectively at coastal, Kenya. Respondent driven sampling, snowball and makeshift methods were used to enroll IDUs and non-IDUs. Convenience and purposive sampling were used to enroll non-DUs from the hospital’s voluntary HIV testing unit. Participant assisted questionnaire was used in collecting socio-demographic data and sexual practices.

**Results:**

Binary logistic regression analysis indicated that higher likelihood of HIV infection was associated with sex for police protection (OR, 9.526; 95% CI, 1.156-78.528; *P* = 0.036) and history of sexually transmitted infection (OR, 5.117; 95% CI, 1.924-13.485; *P* = 0.001) in IDUs; divorced, separated or widowed marital status (OR, 6.315; 95% CI, 1.334-29.898; *P* = 0.020) in non-IDUs; and unemployment (OR, 2.724; 95% CI, 1.049-7.070; *P* = 0.040) in non-drug users. However, never married (single) marital status (OR, 0.140; 95% CI, 0.030-0.649; *P* = 0.012) was associated with lower odds for HIV infection in non-drug users.

**Conclusion:**

Altogether, these results suggest that socio-demographic and sexual risk factors for HIV transmission differ with drug use status, suggesting targeted preventive measures for drug users.

## Background

Consumption of psychoactive substances is an increasing public health problem in the world [1]. An estimated 250 million people between the ages of 15 and 64 years used an illicit drug in 2014 with at least 29 million suffering drug use disorders [2]. Both injection and non-injection substance consumption constitute the global burden of substance use with Africa having as estimated 28 million substance users [2]. The burden of substance use in Africa is compounded by the increasing availability of injection illicit drugs such as heroin, cocaine and methamphetamine especially in peri-urban and urban settings [3–5]. Kenya, like other countries in Africa, is experiencing an alarming increase in the burden of drug use with about 37.1% of the population reporting having used a substance in their life time [6].

Social demographic factors such as the level of education, gender, income and marital status are primary determinants of the health status of drug users [7]. These factors indirectly influence individual drug-use behavior including sharing of needles and soliciting for sex in exchange for drugs or police protection [8, 9]. Directly, these factors predict familial support, nutritional status, adherence to medication and recovery from drug use or addiction [10–12]. Globally, drug-related activity has been associated with age, low level of education, familial dysfunction, unemployment, poverty, drug-related violence and gang activity [13, 14].

Despite availability of antiretroviral (ARV) treatments, HIV infection remains a major public health problem especially in Sub-Saharan Africa. This burden has been partly attributed to recreation drug use which increases the risk of HIV infection and poor adherence to ARVs [11, 15]. At the end of 2015, approximately 36.7 million people were living with HIV/AIDS in the world. During the same period it is estimated that around 13 million people injected drugs of whom 1.7 million were living with HIV. An estimated 70% of people living with HIV infection reside in sub-Saharan Africa [16, 17]. In Kenya, 1.5 million people consisting of 18,327 injection substance users were living with HIV as at the end of 2016. The current prevalence of HIV in injection substance users in the country stands at 18.3% which is higher than that in the general population [18].

Majority of drug users transit from use of non-injection to injection substances or simultaneously use both substances [19–21]. Furthermore, substance consumption differentially predicts HIV infection. For example, previous studies in Texas, USA and China showed that injection substance users have an increased risk of HIV infection in comparison to non-injecting drug users [22, 23]. In Kenya, predictors of HIV in the general population include low income, marital separation, sex work, exposure to sexually transmitted infections (STIs) and gender [24]. In the injection substance using population, high risk sexual practices, exposure to STIs, sexual violence, sharing of needles and syringes as well as poly-substance use are associated with higher rates of HIV infection [4, 25]. Elsewhere, alcohol, khat, marijuana and methamphetamine use are associated with HIV in non-injection substance users [26–28]. However, in Kenya and by extension Africa, information on comparative socio-demographic factors that predict HIV trends in IDUs, non-IDUs and non-drug users is scantly documented. This study therefore sought to fill this gap by investigating the socio-demographic patterns and sexual practices predicting HIV infection among IDUs in comparison to non-IDUs and non-DUs in Coastal Kenya.

## Methods

### Study design, setting and participants

This cross-sectional descriptive study was conducted in July, 2012 - February, 2013 in Mombasa city, Coastal Kenya. Upon obtaining written informed consent, 451 participants were enrolled of which 196 (HIV-infected, *n* = 157 and -uninfected, *n* = 39) were injection drug users, 65 non-injection drug users (HIV-infected, *n* = 17 and -uninfected, *n* = 48) and 190 non-drug users (HIV-infected, *n* = 135 and *n* = 55 -uninfected). Several sampling methods were used as these findings are part of a larger study. The IDUs and non-IDUs were recruited via respondent driven sampling (RDS), snowball and makeshift outreach methods.

Recruitment using RDS was initiated with three seeds who were known drug users from the drug users’ receiving addiction counseling at Bomu hospital. Each seed was given three uniquely coloured coupons with which they used to recruit peers within Mombasa County. These recruited peers were considered as the first wave of participants. Each participant in the first wave who completed the interview was then provided six coupons with which to recruit their peers. Interviews were conducted by research assistants who are trained community health workers. Successive waves of recruitment continued until the waves died (no more participants were being enrolled) before the desired sample size was achieved. At this stage only 148 IDUs and non-IDUs had been recruited. Snowball sampling and makeshift outreach methods were then used to directly recruitment from drug havens by a rehabilitated former injection drug user 113 IDUs and non-IDUs. Convenience and purposive samplings were used to recruit non-drug users who were drop in clients in the hospitals’ HIV voluntary counseling and testing unit. Interviews were conducted by trained community health workers.

The current injection drug users were defined as persons who injected for recreational purpose at least once a day regularly, at the time of the study. Observation of the needle scars was a necessary criterion for their enrollment. The non-IDUs were persons who had never injected drugs during their lifetime but were using one or more non-injection drugs listed in the world drug report [2]. Non-drug users were persons who had never used either injection or non-injection drugs in their lifetime [2]. In addition, eligibility criteria were limited to people who at the time of recruitment were 18 years and above, lived in Mombasa and had provided written informed consent.

### Demographic and sexual practice information

Pilot testing was carried out at Bomu Wema Centre, an outreach clinic in Kisauni sub-county, Mombasa city. Participant assisted questionnaire was administered in English or Swahili by trained community health workers at Bomu Hospital in Changamwe sub-county, Mombasa city. This questionnaire was used to capture the following socio-demographic characteristics; gender, age, level of education, marital status, religion, occupation and income per month. Using the same tool, sexual orientation, age of sexual debut, number of sexual partners, having had unprotected sex, having had sex for police protection or drugs and history of STI was captured.

### HIV testing

HIV testing was performed by trained VCT counselors using Determine™ (Abbott Laboratories, Tokyo, Japan) and Unigold™ (Trinity Biotech Plc, Bray, Ireland) and the results were communicated back within 5 to 10 min. Study participants with positive results for both Determine and Unigold were considered HIV infected based on the Kenya National Guidelines for HIV Testing and Counseling [29].

### Ethical considerations

The study was conducted according to the Helsinki declaration and ethical approval was obtained from the Kenyatta University Ethics Review Committee. Participation in the study was voluntary and written informed consent was obtained from the subjects prior to enrolment in to the study. Confidentiality of participants was observed throughout the study and all participants received free health education on sexually transmitted infections and HIV.

### Statistical analyses

Data analyses were conducted using SPSS, version 20 (IBM SPSS Inc., New York, USA). Pearson’s Chi-square test was used determine the distribution of proportions of independent variables among the HIV infected and uninfected study groups. Age was summarized as medians and compared amongst the study groups using Kruskal Wallis test followed by Dunns post-hoc corrections. Subsequently, binary logistic regressions were performed to identify those factors that were independently associated with HIV infection within IDUs, non-IDUs and non-drug users. In these analyses, variables previously associated with illicit drug use and/or HIV infection in Kenya like education, marital status, religion, occupation, income, sexual orientation, age of sex debut, number of sexual partners, unprotected sex, sex for police protection, sex for drugs and history of STIs [4, 25, 30, 31], including those that showed *P* < 0.1 in the chi-square analyses were entered as independent predictor variables, and HIV status as dependent variable controlling for age and gender. All tests were two-tailed and *P* < 0.05 was considered statistically significant.

## Results

### Socio-demographic characteristics

Table 1 shows the socio-demographic characteristics of the participants. Median age, primary or lack of education, marital separation, Islam religion and earning an income of more than US$ 172.8 were significantly higher among HIV infected injection drug users relative to non-injection drug users and non-drug users (*P* < 0.0001 for all comparisons). In addition, the proportions of sex work and entertainment or beauty therapy occupations were higher in injection drug users in comparison to non-injection drug users and non-drug users.

**Table 1:** Socio-demographic characteristics of the study participants

Characteristic	Non-DU		Non-IDU		IDU		P
	HIV[−], *n* = 135	HIV[+], *n* = 55	HIV[−], *n* = 48	HIV[+], *n* = 17	HIV[−], *n* = 39	HIV[+], *n* = 157	
Female	75 (55.6)	29 (52.7)	19 (39.6)	6 (35.3)	14 (35.9)	86 (54.8)	0.081
Median age (IQR), yrs.	30.0 (14.3)	41.7 (14.2)	30.4 (11.8)	30.4 (11.0)	26.8 (5.2)^a^	30.6 (6.5)	**< 0.0001**
Education							
Secondary or college	74 (54.8)	25 (45.4)	22 (45.9)	6 (35.3)	12 (30.8)	35 (22.3)	**< 0.0001**
Primary or none	61 (45.2)	30 (54.6)	26 (54.2)	11 (64.7)	27 (69.3)	122 (76.7)	
Marital status							
Married	59 (43.7)	28 (50.9)	23 (47.9)	5 (29.4)	9 (23.1)	17 (10.8)	**< 0.0001**
Never married (single)	52 (38.5)	2 (3.6)	12 (25.0)	2 (11.8)	20 (51.3)	44 (28.0)	
Divorced, separated or widowed	24 (9.6)	25 (45.5)	13 (27.2)	10 (58.8)	10 (25.6)	96 (61.1)	
Religion							
Catholic	50 (37.0)	11 (20.0)	18 (37.5)	5 (29.4)	11 (28.2)	43 (27.4)	**< 0.0001**
Protestant	54 (40.0)	33 (60.0)	18 (37.5)	6 (35.3)	5 (12.8)	36 (22.9)	
Muslim	31 (23.0)	11 (20.0)	12 (25.0)	6 (35.3)	23 (59.0)	78 (49.7)	
Occupation							
CSW	0 (0.0)	0 (0.0)	0 (0.0)	0 (0.0)	6 (15.4)	35 (22.3)	–
Entertainment or beauty therapy	5 (3.7)	3 (5.5)	3 (6.3)	2 (11.8)	3 (7.7)	41 (26.1)	
Transport	12 (8.9)	6 (10.9)	9 (18.8)	4 (23.5)	13 (33.3)	35 (22.3)	
Hospitality	7 (5.2)	3 (5.5)	8 (16.7)	2 (11.8)	3 (7.7)	10 (6.4)	
Small business	35 (25.9)	25 (45.5)	13 (27.1)	5 (29.4)	12 (30.8)	27 (17.2)	
Service	26 (19.3)	11 (20.0)	10 (20.8)	2 (11.8)	2 (5.1)	4 (2.5)	
Unemployed	50 (37.0)	7 (12.7)	5 (10.4)	2 (11.8)	0 (0.0)	5 (3.2)	
Income, Kshs/month^a^							
> 15,000	27 (20.0)	13 (23.6)	13 (27.1)	3 (17.6)	26 (66.7)	127 (80.9)	**< 0.0001**
5000-15,000	48 (35.6)	22 (40.0)	22 (45.8)	7 (41.2)	11 (28.2)	25 (15.9)	
< 5000	60 (44.4)	20 (36.4)	13 (27.1)	7 (41.2)	2 (5.1)	5 (3.2)	

Data are presented as number and proportions (%) of subjects, unless otherwise indicated. Statistical analyses were conducted using the Pearson’s chi-square. Non-DU, non-drug users. *Non-IDU* non-injection drug users, *IDU* injection drug users. ^a^As at June 30th 2016, US$ was equivalent to KShs 86.8 ^a^*P* < 0.01 vs. HIV-infected and uninfected IDUs and non-DUs. Values in bold indicate significant *P*-values

### Sexual practices

The proportions of participants reporting early age sexual debut, > 1 sexual partners, unprotected sex and history of sexually transmitted infections (all *P* < 0.0001) was significantly higher in HIV-infected injection drug users than in non-injection drug users and non-drug users (Table 2). Likewise, the frequency of bisexuality, homosexuality, sex for police protection as well as sex for drugs was higher in HIV-infected injection drug users as compared to non-injection drug users and non-drug users (Table 2).

**Table 2:** Sexual practices and history of sexually transmitted infections

Characteristic	Non-DU		Non-IDU		IDU		P
	HIV[−], *n* = 135	HIV[+], *n* = 55	HIV[−], *n* = 48	HIV[+], *n* = 17	HIV[−], *n* = 39	HIV[+], *n* = 157	
Sexual orientation							
Bisexual	1 (0.7)	0 (0.0)	0 (0.0)	0 (0.0)	2 (5.1)	13 (8.3)	–
Heterosexual	134 (99.3)	55 (100.0)	47 (97.9)	17 (100.0)	35 (89.7)	131 (83.4)	
Homosexual	0 (0.0)	0 (0.0)	1 (2.1)	0 (0.0)	2 (5.1)	13 (8.3)	
Age of sex debut < 15 years	36 (26.7)	10 (18.2)	13 (27.1)	5 (29.4)	8 (20.5)	76 (48.4)	**< 0.0001**
No. of sexual partners							
0	25 (18.5)	21 (38.2)	10 (20.8)	4 (23.5)	3 (7.7)	5 (3.2)	**< 0.0001**
1	85 (63.0)	27 (49.1)	28 (58.3)	9 (52.9)	17 (43.6)	49 (31.2)	
> 1	25 (18.5	7 (12.7)	10 (20.8)	4 (23.5)	19 (48.7)	103 (65.6)	
Unprotected sex	14 (10.4)	7 (12.7)	14 (29.2)	5 (29.4)	7 (17.9)	49 (31.2)	**< 0.0001**
Sex for police protection	0 (0.0)	1 (1.8)	0 (0.0)	0 (0.0)	1 (2.6)	34 (21.7)	–
Sex for drugs	0 (0.0)	0 (0.0)	0 (0.0)	1 (5.9)	7 (17.9)	39 (24.8)	–
History of STI	52 (38.5)	31 (56.4)	26 (54.2)	8 (47.1)	9 (23.1)	102 (65.0)	**< 0.0001**

Data are presented as number and proportions (%) of subjects. Statistical analyses were conducted using the Pearson’s chi-square. Non-DU, non-drug users. *Non-IDU* non-injection drug users. *IDU* injection drug users, *STI* sexually transmitted infection. Values in bold indicate significant *P*-values

### Association of socio-demographic characteristics, sexual practice and HIV status

To identify the predictors of HIV infection, binary logistic regression was conducted separately for each study group (injection drug users, non-injection drug users and non-drug users) such that all HIV infected individuals were modeled against all of the HIV negative individuals controlling for age and gender. Data were presented as odds ratios and 95% confidence intervals (Table 3). These analyses illustrated that HIV infection was associated with divorced, separated or widowed marital status (OR, 2.768; 95% CI, 0.888-8.633; *P* = 0.079); small businesses (OR, 2.454; 95% CI, 0.944-6.376; *P* = 0.065); age of sex debut < 15 years (OR, 2.547; 95% CI, 0.965-6.723; *P* = 0.059) and > 1 sexual partners (OR, 2.021; 95% CI, 0.925-4.415; *P* = 0.078); sex for police protection (OR, 9.526; 95% CI, 1.156-78.528; *P* = 0.036) and history of sexually transmitted infection (OR, 5.117; 95% CI, 1.924-13.485; *P* = 0.001) among injection drug users; divorced, separated or widowed marital status (OR, 6.315; 95% CI, 1.334-29.898; *P* = 0.020) in non-injection drug users; and unemployment (OR, 2.724; 95% CI, 1.049-7.070; *P* = 0.040) and history of sexually transmitted infections (OR, 1.838; 95% CI, 0.909-3.717; *P* = 0.090) in non-drug users. In contrast, single marital status (OR, 0.140; 95% CI, 0.030-0.649; *P* = 0.012) was linked to HIV infection amongst non-drug users.

**Table 3:** Socio-demographic and sexual practices associated with HIV infection

Characteristic	Non-DU		Non-IDU		IDU	
	OR (95% CI)	*P*-value	OR (95% CI)	*P*-value	OR (95% CI)	*P*-value
Education						
Secondary or college	Ref		Ref		Ref	
Primary or none	1.042 (0.520-2.089)	0.907	1.710 (0.512-5.711)	0.383	1.499 (0.647-3.475)	0.345
Marital status						
Married	Ref		Ref		Ref	
Never married (single)	0.140 (0.030-0.649)	0.012	0. 926 (0.135-6.336)	0.938	0.827 (0.290-2.360)	0.723
Divorced, separated or widowed	1.804 (0.810-4.016)	0.149	6.315 (1.334-29.898)	0.020	2.768 (0.888-8.633)	0.079
Religion						
Catholic	Ref		Ref		Ref	
Protestant	0.715 (0.256-2.000)	0.523	1.216 (0.312-4.734)	0.778	1.230 (0.365-4.146)	0.738
Muslim	1.858 (0.749-4.610)	0.182	1.753 (0.426-7.221)	0.437	0.866 (0.343-2.183)	0.760
Occupation^a^						
CSW	…		0.992 (0.932-1.057)	0.806	1.443 (0.471-4.424)	0.521
Entertainment or beauty therapy	1.483 (0.309-7.118)	0.622	2.351 (0.325-17.002)	0.397	2.790 (0.699-11.138)	0.146
Transport	0.838 (0.263-2.667)	0.764	1.305 (0.328-5.201)	0.706	1.196 (0.440-3.259)	0.725
Hospitality	0.718 (0.160-3.233)	0.666	1.414 (0.251-7.962)	0.695	1.285 (0.291-5.683)	0.741
Small business	0.561 (0.275-1.142)	0.111	0.920 (0.267-3.165)	0.894	2.454 (0.944-6.376)	0.065
Service	1.241 (0.515-2.993)	0.631	0.467 (0.089-2.466)	0.370	3.352 (0.452-24.820)	0.236
Unemployed	2.724 (1.049-7.070)	0.040	1.179 (0.202-6.885)	0.855	…	…
Income, US$/mos.						
> 172.8	Ref		Ref		Ref	
57.6-172.8	0.883 (0.361-2.161)	0.785	1.498 (0.318-7.053)	0.609	0.540 (0.213-1.367)	0.193
< 57.6	0.804 (0.318-2.035)	0.646	2.686 (0.528-13.676)	0.234	0.893 (0.127-6.255)	0.909
Sexual practices^a^						
Homosexual or bisexual	…	…	…	…	2.501 (0.726-8.611)	0.146
Age of sex debut < 15 years	1.254 (0.539-2.919)	0.599	1.183 (0.325-4.310)	0.799	2.547 (0.965-6.723)	0.059
> 1 sexual partners	1.261 (0.461-3.392)	0.660	1.116 (0.290-4.292)	0.873	2.021 (0.925-4.415)	0.078
Unprotected sex	2.134 (0.738-6.168)	0.162	1.053 (0.305-3.636)	0.935	0.772 (0.276-2.160)	0.621
Sex for police protection	…	…	…	…	9.526 (1.156-78.528)	0.036
Sex for drugs	…	…	…	…	1.159 (0.437-3.073)	0.767
History of STI	1.838 (0.909-3.717)	0.090	1.407 (0.422-4.694)	0.578	5.117 (1.924-13.485)	0.001

Binary logistic regression was conducted separately for each study group (IDU, non-IDU and non-DU) such that all HIV infected individuals were modeled against all of the HIV negative individuals controlling for age, and gender. Anon-practitioners of the listed occupations or practices were used as the reference category. Data are presented as odds ratios (OR) and 95% confidence interval (CI). *IDU* injection drug users, *Non-DU* non-drug users, *CSW* commercial sex worker, *STI* sexually transmitted infection

## Discussion

In the current study, we show that HIV infected individuals were at least 30 years old among the injection drug users, suggesting that older age may be a risk factor of HIV infection in this cohort of Kenyan injection drug users. This finding is similar to previous prospective studies in China and Canada showing that an age of at least 25 years predicts HIV infection [32, 33]. In part, this suggests that increasing exposure to sexual behavioral and injection risk practices increases with age among injection drug users. This hypothesis is consistent with previous studies among Chinese injection drug users showing that HIV positivity was associated a longer history of injection drug use and experience of needle and syringe sharing [22]. Strategies aimed at harm reduction, for example, through provision of free needles and safe commercial sexual practices may, therefore, lower HIV infection rates among injection substance using communities.

The higher rates of low education of at least 70% in both HIV infected and uninfected injection drug users, suggest that education is an important determinant of substance use. These results are in part comparable to recent studies in China showing that lower education levels are associated with unsafe injection practices in injection drug users [34] and results of a drug addiction treatment study in Iran indicating that at least 60% of drug users were having less than high school education [35]. Little or no education reflects low awareness on the effects of drug consumption, initiation into drug use, risk factors and safe injection practices. In support of this fact, previous studies show that school drop-out predicts initiation into drug use [35, 36]. Additionally, low education among injection drug users indicates a likelihood of needle sharing and non-participation in HIV interventions [37–39].

Consistent with previous studies in the Iran, USA and Taiwan showing that marital separation, divorce and widowhood were associated with increased risk of HIV infection in injection drug users [40–42]. The rates of individuals reporting being divorced, separated or widowed were high among all the infected groups. In regression analyses controlling for confounders, these individuals were still more likely to be HIV infected in the injection and non-injection drug users. The divorced, widowed or separated individuals form one of the high HIV risk groups in Kenya [43]. However, it is not known whether these individuals acquire the infections pre- and/or post-separation of the marital unions. A number of interacting factors may expose such persons to risk of getting infected. For instance, following loss of a marital partner and loss of income, an individual may choose occupations that increase risk of infection. In contrast, individuals reporting never married marital status were fewer in the infected study groups and were less likely to be HIV infected in the non-drug users. These results are, in part, similar to retrospective demographic and health surveys across Africa showing that married, widowed and divorced women were more likely to be HIV positive compared to never married women [44]. While the underlying reasons for these lower rates remain to be elucidated it is likely that this group is younger, more educated and hence more informed about HIV risk and preventive measures. This assertion is partly supported by previous studies in Tanzania showing that single women with secondary or higher education were less likely to have multiple sexual partners and use condoms than married women with no education [45, 46].

Although Muslim religious affiliation was not independently associated with HIV infection in the regression analyses; Muslims accounted for a majority of HIV infected (49.7%) and uninfected (59.0%) injection substance users. Consistent with the high frequency of Muslims in the HIV infected and uninfected substance users also had higher rates of primary or below primary education levels of 76.7% and 69.3%, respectively. These figures are comparable to results of previous surveys illustrating higher prevalence (69%) of primary or lack of primary education among the Muslim communities at coastal Kenya [47]. In fact, a lack of education, primary education and failure to complete high school are linked to illicit drug use or injection equipment sharing at several coastal Kenya cities [29, 35, 48, 49]. This supports a paradigm of low levels or lack of education driving injection substance among the Muslim communities at coastal Kenya.

It was noted that the frequency of small business and unemployment were, respectively, fewer in HIV-infected non-drug users and injection drug users. But in regression models adjusting for confounders, engaging in small business and being unemployed were associated with likelihood of HIV-infection in non-drug users and injection drug users, respectively. All these scenarios point to involvement in low income occupations as the main contributor to risk of HIV infection. This is supported by previous studies in Canada showing associations between stimulant drug use and engaging in low-threshold employment [50] and studies in Spain showing higher prevalence of HIV infection among unemployed injection drug users [51]. This result may be related to likely additional engagement in secondary occupations to supplement income such as sex for cash. Individuals in small business and unemployed injection drug users may thus be targeted for spreading HIV prevention social marketing interventions [52]. The finding of more individuals with higher income levels in both HIV-infected and -uninfected injection drug users, partly relates to class subsets in the drug injection communities at this coastal region of Kenya. In addition, studies among injection drug users in the United States indicate that income is a key determinant of low-to-high use and low-to-high risk classes of injection drug users [53]. Subsequently, income levels may determine the frequency of injection, affordability of drug injection equipment and involvement in risky sexual practices. Therefore, we advocate for the integration of income generating programs into the intervention and rehabilitation packages of injection drug users to improve their quality of life and prevent reversion.

Nearly 50 % of the injection drug users reporting sex debut at age less than 15 years were HIV infected. These results remained predictive of the HIV infection in injection drug users even after controlling for confounders in the regression analyses. This finding differs from data from demographic and health surveys conducted from Uganda, Tanzania, South Africa, Zimbabwe and Malawi showing that age to first sex ranged from 17 to 19 years and 16-19 years for males and females, respectively [54]. Age is an important component of sex and reproductive health of a person. At a young age an individual is still a minor and incapable of making informed decisions regarding safer sex. Such underage persons are also prone to sexual and/or physical abuse that is frequently associated with risk of HIV acquisition. According to the Kenya 2012 HIV risk estimates, 21% of young adults aged 15 to 24 years reported sexual debut before 15 years of age [43] compared to 48.4% in HIV-infected injection drug users in the current study. The interrelationships of these factors mirror previous studies in Russia illustrating that age at first drink is associated with multiple sex partners and age of sex debut [55]. Young people in Mombasa may thus be initiating substance use and sex work at a young age to escape ravages of poverty but exposing to risk of infection.

Having more than one sexual partner and engaging in sex for police protection were identified as important sexual practices predicting HIV infection among injection drug users. These findings are consistent with previous studies in the same area showing that injection drug users report multiple past and new sex partners [36] and studies in South Africa showing complex interacting economies of drugs and sex work whereby sex was exchanged for drugs [56]. Multiplicity of sexual partners often increases the risk of acquiring sexually transmitted infections including HIV [17, 40]. While this finding is consistent with the results of the Kenya AIDS Indicator Survey, 2012 for the general population [43] similar studies have shown associations between multiple sexual partnerships and trading sex for drugs in HIV positive Tanzanian heroin users [8]. For the first time we show that sex for police protection was associated with higher likelihood of having HIV infection in this Kenyan cohort of injection drug users. These findings mirror previous studies in Pakistan indicating that harassment, abuse and exploitation of vulnerable groups (including injection drug users, sex workers and men who have sex with men). These violations frequently result from non-state actors such as relations and sex worker clients, and state actors such as the police [57]. In the current study, it appears that injection drug users submit to sex for police protection to avoid arrest and imprisonment, hence, targeted programs for HIV prevention should also include the police and other state actors.

Consistent with previous studies [22, 58] higher rates of unprotected sex and a history of sexually transmitted infections were associated with HIV infection among injection drug users. These results suggest that high risk sexual practices especially while intoxicated exposes to unlimited risk of HIV transmission. Previous studies among Iranian injection drug users in harm-reduction drop-in centers and Brazilian crack users indicated that unsafe sex risk practices including unprotected sex are associated with risk of HIV infection [59, 60]. Likewise, previous studies in the US showed that injection drug use is associated with high risk of sexually transmitted infections [40]. We, therefore, advocate for programs targeting behavioral change and treatment of sexually transmitted infections in curbing the high HIV menace among the injection drug users.

## Conclusion

In summary, we found that multiple risk factors contribute to HIV infection. Predictors of HIV infection in this cohort of coastal Kenyan injection drug users include sex for police protection and history of sexually transmitted infections. Among non-IDUs marital separation predicted HIV infection while having never been married, being unemployed and a history of STIs were predictive of HIV status among the non drug users. Since the study design was cross-sectional, it is difficult to know the time-point of HIV contact. A longitudinal approach would therefore yield more detailed information on sexual practices link to acquisition of HIV infection. Furthermore, self-reported exposure to sexually transmitted infections and sexual practices may be confounded by recall bias. Moreover, drug use was based on self-reported history which is prone to social desirability bias.

We recommend future studies to confirm drug use via appropriate testing methods like hair, saliva and urine analysis. We recommend similar studies to be replicated in other parts of the country where studies based on self reported drug use have been carried out before. Since drug use is a socio-medical problem, we recommend interventions that are anchored on a multi-sectoral approach. This approach need to encompass improvement in school enrollment and retention, upscale drug prevention awareness campaigns, psycho-therapy, treatment, support groups as well as financial empowerment. In addition, as part of injection drug use targeted interventions for state actors (lawmakers, law enforcers and criminal justice authorities) should be provided with educational programs on human rights, safety and public health interventions.
